# Clinical Examination of the Urine and Urinary Diagnosis

**Published:** 1910-02

**Authors:** 


**Clinical Examination of the Urine and Urinary Diagnosis.** By **J. Bergen Ogden,** M.D., Medical Chemist to the Metropolitan Life Insurance Company, New York. Third edition, revised. Octavo of 427 pages, illustrated. Philadelphia and London: W. B. Saunders Company. 1909. (Cloth, $3.00 net.)

A third edition of this work has been called for, the two preceding editions having been exhausted. Changes incident to advancing knowledge on the subject have been made in this revision, by the omission of some matter not now considered of clinical importance, and by the addition of newer ideas brought about by later study and investigation.

The great aim of practical medicine is the cure of disease. Enlightened therapeutics must be preceded by a correct diagnosis in order that proper methods may be applied in a scientific manner. As an aid in bringing the entire picture into view this book is helpful. J. A. R.

